# The disparity of measuring bone mineral content using bioimpedance and dual-energy absorptiometry in the context of hyperparathyroidism

**DOI:** 10.1590/2175-8239-JBN-2020-0063

**Published:** 2020-08-31

**Authors:** Shirley Ferraz Crispilho, Eduardo Jorge Duque, Kalyanna Soares Bezerra, Rosa Maria R. Pereira, Vanda Jorgetti, Rosilene M. Elias, Rosa M. A. Moysés

**Affiliations:** 1 Universidade Nove de Julho, São Paulo, SP, Brasil.; 2 Universidade de São Paulo, Faculdade de Medicina, Hospital das Clínicas, São Paulo, SP, Brasil.

**Keywords:** Impedance, Absorptiometry, Photon, Body Composition, Chronic Kidney Disease-Mineral and Bone Disorder, Osteoporosis, Phosphate, Hyperparathyroidism, Impedância, Absorciometria de Fóton, Composição corporal, Distúrbio Mineral e Ósseo na Doença Renal Crônica, Osteoporose, Fosfato, Hiperparatireoidismo

## Abstract

**Introduction:**

Body composition is critical for the evaluation of patients with Chronic Kidney Disease (CKD) and can be obtained from either multifrequency bioelectrical impedance analysis (BIA) or dual-energy absorptiometry (DXA). Although the discrepancy between the results obtained from both methods has already been described, reasons are unknown, and might be related to secondary hyperparathyroidism, which is associated with bone loss.

**Methods:**

We have evaluated 49 patients (25 males and 24 females): 20 with CKD not on dialysis and 29 on maintenance hemodialysis [18 with severe hyperparathyroidism (HD-SHPT) and 11 submitted to parathyroidectomy (HD-PTX)]. All patients underwent DXA and BIA.

**Results:**

The median age and body mass index (BMI) were 49 years and 25.6 kg/m^2^, respectively. Patients exhibited low bone mineral content (BMC) measured by DXA, particularly those from the HD-SHPT group. The largest BMC measurement disagreement between DXA and BIA was found in the HD-SHPT group (p=0.004). Factors independently associated with this discrepancy in BMC measurement were serum phosphate (p=0.003) and patient group (p=0.027), even after adjustments for age, BMI, and gender (adjusted r2=0.186). PTX attenuated this difference.

**Discussion:**

BIA should be interpreted with caution in patients with SHPT due to a loss of accuracy, which can compromise the interpretation of body composition.

## Resumo

**Introdução:**

A composição corporal é fundamental para a avaliação de pacientes com Doença Renal Crônica (DRC), e pode ser obtida por análise de impedância bioelétrica por multifrequência (BIA) ou absorciometria de dupla energia (DXA). Embora a discrepância entre os resultados obtidos pelos dois métodos já tenha sido descrita, os motivos são desconhecidos e podem estar relacionados ao hiperparatireoidismo secundário, devido à perda óssea.

**Métodos:**

Avaliamos 49 pacientes (25 homens e 24 mulheres): 20 com DRC não em diálise e 29 em hemodiálise de manutenção [18 com hiperparatireoidismo grave (HD-SHPT) e 11 submetidos à paratireoidectomia (HD-PTX)]. Todos os pacientes foram submetidos à DXA e BIA.

**Resultados:**

A mediana da idade e do índice de massa corporal (IMC) foram de 49 anos e 25,6 kg/m^2^, respectivamente. Os pacientes exibiram baixo conteúdo mineral ósseo (CMO) medido pelo DXA, particularmente aqueles do grupo HD-SHPT. A maior discordância da medida do CMO entre DXA e BIA foi encontrada no grupo HD-SHPT (p = 0,004). Os fatores independentemente associados a essa discrepância na medida do CMO foram fosfato sérico (p = 0,003) e grupo de pacientes (p = 0,027), mesmo após ajustes para idade, IMC e sexo (r2 ajustado = 0,186). PTX atenuou essa diferença.

**Discussão:**

A BIA deve ser interpretada com cautela em pacientes com HPTS devido a uma perda de precisão, o que pode comprometer a interpretação da composição corporal.

## Introduction

Assessment of nutritional and hydration status is important in patients with chronic kidney disease (CKD). Body composition analysis can be obtained through either multifrequency bioelectrical impedance (BIA) or dual energy absorptiometry (DXA)^1^. Since BIA is currently the most available and less expensive method, this technique, rather than DXA, has been applied to most of our patients. However, some authors have shown a significant disagreement between these methods, particularly for the bone mineral content (BMC) measurement^2^^-^ 4. Whereas BMC is measured by DXA, it is predicted by BIA using equations^5^. BIA yields a less consistent estimation of BMC, overestimating it, which makes this method not recommended for identifying patients at great risk of fracture. Although the discrepancy between BIA and DXA measurements has already been described, reasons have still not been elucidated. Disorders of mineral and bone metabolism in CKD (CKD-MBD) involve the participation of a series of events including increase of serum levels of phosphate, fibroblast growth factor 23 and parathyroid hormone (PTH), and reduction of serum calcium and 25-vitamin D that are usually associated with a progressive bone loss and high risk of fractures^6^. Secondary hyperparathyroidism (SHPT) is a highly prevalent CKD-MBD disorder in patients with advanced CKD leading to bone loss and fracture risk. Age and duration of CKD are other factors associated with bone loss in this population^7^.

It has been previously demonstrated that patients on dialysis with SHPT exhibited a higher disagreement between DXA and BIA measurements than those on conservative management^3^. Nevertheless, the design of that study did not allow us to conclude whether the disagreement was a result of the CKD duration or the presence of SHPT. Trying to answer this question, in the current study we compared results obtained from DXA and BIA in patients with CKD on conservative management and patients with CKD on hemodialysis with and without SHPT.

## Methods

We evaluated 20 patients with CKD on conservative management and 29 on hemodialysis (18 with severe hyperparathyroidism - HD-SHPT - and 11 already submitted to parathyroidectomy at least 1 year prior to our analysis - HD-PTX). Total body composition was determined using BIA and DXA (Hologic QDR 4500A; Hologic Inc. Bedford, MA, USA). BIA was performed using a segmental tetrapolar bioelectrical impedance in all patients while recumbent, by the multifrequency InBody™ S10 (Biospace Co.,Ltd., Korea) device. The following parameters were evaluated: bone mineral content (BMC), fat mass (FAT), and lean mass (LM).

## Results

The characteristics of patients according to each group are described in Table 1. Patients from the HD-SHPT group were less heavy and had a lower dialysis vintage than those from the HD-PTX group. They also presented higher phosphate, alkaline phosphatase (AP), and parathormone (PTH).

**Table 1:** Patients' characteristics according to group: chronic kidney disease (CKD), patients with secondary hyperparathyroidism on hemodialysis (HD-SHPT), and patients on hemodialysis submitted to parathyroidectomy (HD-PTX).

	CKD	HD-SHPT	HD-PTX	p
Age (y)	52.5 ± 14.3	41.6 ± 14.9	44.9 ± 13.4	0.06
Male gender (%)	50	50	54.5	0.81
eGFR, mL/min/1.73m^2^	47.3 ± 10.2	N/A	N/A	N/A
Dialysis vintage (yrs)	N/A	6.8 (4, 9.3)*	13 (8, 21)	0.002
BMI (kg/m^2^)	27.1 ± 3.8	23.7 ± 4.1*	30.3 ± 12.0	0.02
Ca (mg/dL)	9.4 (9.2, 10.1)	9.5 (8.7, 10.0)	8.9 (8.3, 10.1)	0.28
P (mg/dL)	3.3 ± 0.6	6.0 ± 1.5#	4.6 ± 1.1	<0.0001
AP (UI/L)	81 (69, 102)	296 (209, 545)#	83 (67, 106)	<0.0001
PTH (pg/mL)	52 (47, 71)	1423 (1099, 1656)#	33 (26, 51)	<0.0001
25(OH) Vitamin D (ng/mL)	24 (22, 32)	27 (19, 33)	29 (24, 39)	0.68

eGFR, estimated glomerular filtration rate; BMI, body mass index; Ca, calcium; P, phosphate; AP, alkaline phosphatase; PTH, parathyroid hormone.

* p <0.05 vs. HD-PTX;

# p < 0.05 vs. CKD and HD-PTX.

Using DXA measurement, women presented higher fat mass (24.2 ± 8.8 vs. 19.1 ± 6.1 kg; p = 0.027) and lower lean mass (41.1 ± 5.8 vs 53.3 ± 9.3 kg; p < 0.0001) and bone mineral content (1.9 ± 0.4 vs. 2.3 ± 0.7 kg; p = 0.0015). However, we found no significant difference according to gender in BMI (25.5 ± 4.2 vs 25.8 ± 3.9 kg/m^2^; p = 0.78) neither in the disagreement of BMC obtained from DXA and BIA (-662 vs -852 g; p = 0.33).

The analysis of DXA results of the 49 patients showed a positive association of BMC with BMI (r = 0.35; p = 0.016) and lean mass (r = 0.71; p < 0.0001), but not with fat mass (r = 0.21; p = 0.17).

**Figure 1:**
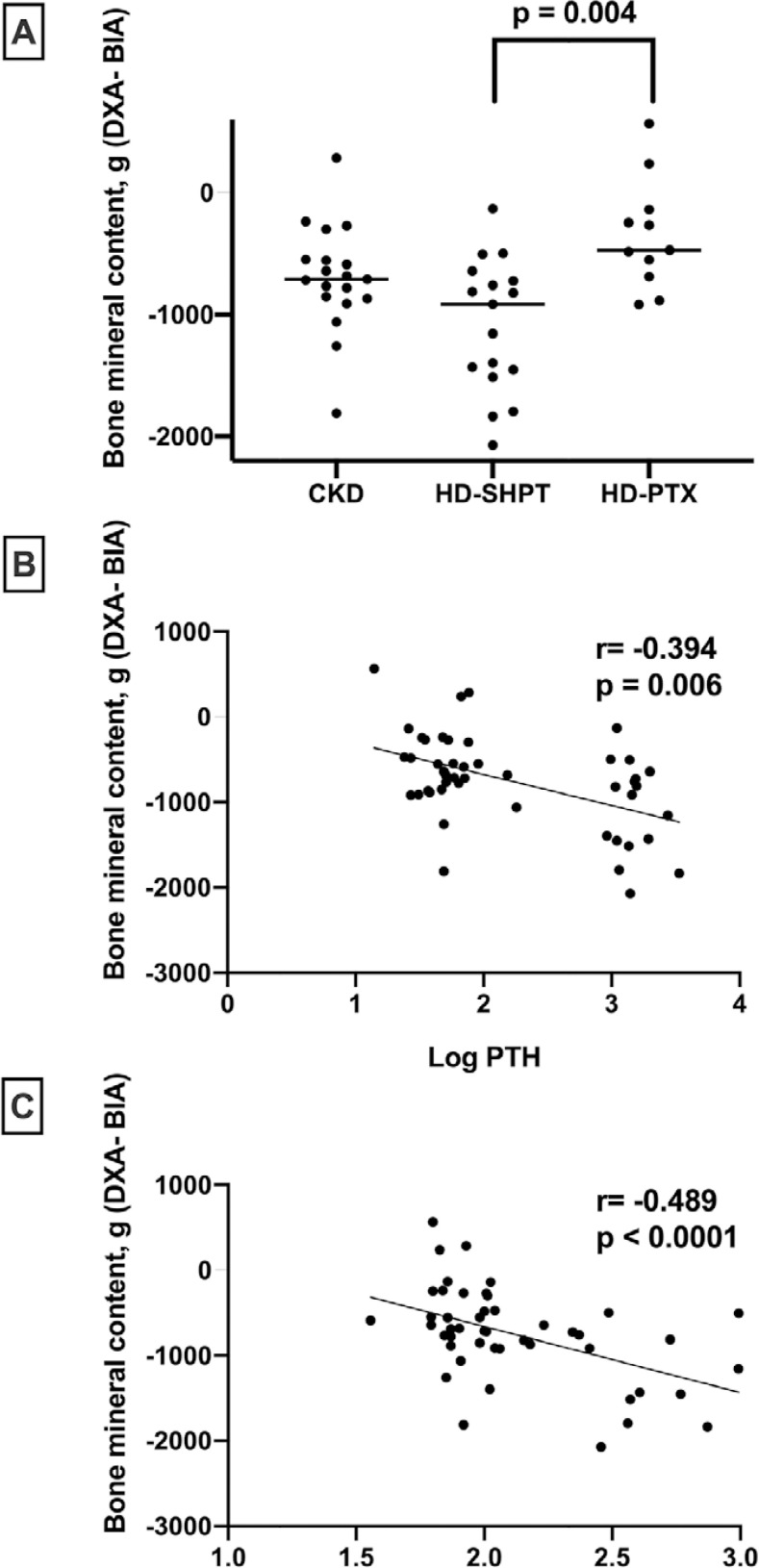
Disagreement between DXA and BIA results across the different groups and its correlation with biomarkers of secondary hyperparathyroidism. A: comparison of (DXA - BIA) bone mineral content (BMC) among the groups showing the higher disagreement in the HD-SHPT group, despite their lower age and dialysis vintage than the HD-PTX group. B: Correlation of the difference of BMC between DXA and BIA with PTH, showing that the higher the PTH, the more significant the difference. C: same finding for AP. PTH = parathormone; AP = alkaline phosphatase

Lean mass measurement was also different using BIA and DXA in the HD-PTX group. We found significant differences between BIA and DXA regarding fat content in both HD-SHPT and HD-PTX patients. Confirming our hypothesis, patients from the HD-SHPT group exhibited lower BMC measured by DXA than that measured by BIA, as shown in Table 2. The largest disagreement of BMC measurements obtained from DXA and BIA was found in the HD-SHPT group (CKD= -711g, 95%CI -851 to -556g; HD-SHPT= -915g, 95%CI -1453 to -724; HD-PTX= -473g, 95%CI -688 to -138g; p= 0.004), as shown in Figure 1A.

**Table 2:** Measurements of body composition according to group: chronic kidney disease (CKD), patients with secondary hyperparathyroidism on hemodialysis (HD-SHPT), and patients on hemodialysis submitted to parathyroidectomy (HD-PTX).

	CKD	HD-SHPT	HD-PTX	p
FAT				
-DXA (kg)	25.7 (17.8-29.5)	17.4 (12.2-24.1)* #	20.0 (14.9-24.2)#	0.049
-BIA (kg)	25.8 (17.0-30.5)	12.7 (7.1-22.1)*	23.2 (17.6- 28.5)	0.012
LEAN				
-DXA (kg)	45.9 (41.1-56.9)	41.9 (38.2-51.0)	47.0 (40.5-58.7)#	0.167
-BIA (kg)	46.9 (41.6-55.8)	41.4 (38.1-53.5)	42.5 (37.5-50.6)	0.496
BMC				
-DXA (kg)	2.27 ± 0.57#	1.81 ± 0.52* #	2.30 ± 0.66#	0.04
- BIA (g)	3.01 ± 0.60	2.90 ± 0.71	2.65 ± 0.47	0.30

DXA, dual-energy absorptiometry; BIA, bioelectrical impedance analysis; BMC, bone mineral content.

* p< 0.05 vs. other groups;

# p < 0.05 vs. BIA in the same group.

There was a significant association between the difference in BMC obtained from DXA and BIA with parathyroid hormone and alkaline phosphatase, as shown in Figures 1B and 1C. We also saw a trend toward an association between the difference in BMC and serum phosphate (r= -0.21; p = 0.15). Factors identified in a multiple regression analysis independently associated with the discrepancy in BMC measurement were serum phosphate (p=0.003) and the group of patients (p=0.027), even after adjustments for age, BMI, and gender (adjusted r^2^=0.186). To further explore whether PTX would influence these results, patients were divided into two groups (with and without PTX). We confirmed that serum phosphate (p=0.018) was positively associated with the discrepancy in BMC measurements whereas PTX (p=0.008) attenuated this difference (adjusted r^2^=0.231).

## Discussion

Our results support the hypothesis that BIA should be interpreted with caution in patients with SHPT since high levels of PTH and AP might lead to greater bone loss and, consequently, to a greater disagreement when compared to DXA. The loss of accuracy of BIA might occur because BMC is not directly measured and is instead obtained using values of fat-free mass in an algorithm developed from normal individuals. Furthermore, the overestimation of BMC is associated with an underestimation of lean mass. This misinterpretation may compromise the management of the nutritional status, as well as of the bone disease, in patients with SHPT.

In addition to the aforementioned effect of SHPT on bone loss, we could confirm that PTX might be beneficial to restore bone mass^8^. The effect of PTX was independent of serum phosphate and contributed to reducing the discrepancy of BMC content measurement obtained from DXA and BIA. In addition, serum phosphate might have an independent deleterious effect on trabecular bone mass. We and other authors have shown that animals fed a high-phosphate diet presented a lower trabecular bone volume. This effect was independent of renal function and serum PTH^9^ ^10^. Although the exact mechanisms by which a high-phosphate diet could impair bone remodeling and volume are not completely understood, it is recognized that hyperphosphatemia is associated with an increase in serum levels of fibroblast growth factor 23 and in bone expression of sclerostin^11^, an inhibitor of bone formation.

In conclusion, we were able to confirm our hypothesis that the more advanced the SHPT, the more severe the bone loss, and the greater the disagreement between DXA and the BIA while measuring BMC.
